# Frequency and patient attributes associated with emergency department visits after discharge: Retrospective cohort study

**DOI:** 10.1371/journal.pone.0275215

**Published:** 2022-10-14

**Authors:** Rita Salgado, Bruno Moita, Sílvia Lopes

**Affiliations:** 1 NOVA National School of Public Health, Universidade NOVA de Lisboa, Lisboa, Portugal; 2 Algarve University Hospital Center, Faro, Portugal; 3 NOVA National School of Public Health, Public Health Research Center, Universidade NOVA de Lisboa, Lisbon, Portugal; 4 Comprehensive Health Research Center, Universidade NOVA de Lisboa, Lisbon, Portugal; Auburn University, UNITED STATES

## Abstract

**Background:**

The utilization of emergency department (ED) during the post-discharge period may provide relevant insights to reduce fragmentation of care, particularly in a context of general intense use. We aimed to describe frequency and patient attributes associated with emergency department (ED) visits within 30 days of inpatient discharge in a Portuguese health region–Algarve.

**Methods:**

Secondary data on inpatient and emergency care, for adult patients discharged in 2016. To analyse the association between outcome–ED visit within 30 days of discharge–and selected variables (admission type and groups of or individual illnesses/conditions), we used age- and sex-adjusted odds ratios (aOR). We included all adult patients (aged ≥18 years) discharged during 2016 from the region’s public hospital inpatient departments. The period for ED visits also included January 2017.

**Results:**

For 21,744 adults discharged in 2016 (mean age: 58 years; 60% female), 23 percent visited ED at least once within 30 days of discharge. Seventy-five percent of those visits were triaged with high clinical priority. Patients with more comorbidities or specific groups of illnesses/conditions had a significant increased risk of returning ED (aOR and 95% confidence intervals–endocrine: 1.566; 1.256–1.951; mental illness: 1.421; 1.180–1.713; respiratory: 1.308; 1.136–1.505).

**Conclusion:**

Patients returned ED after inpatient discharge frequently and for severe reasons. Patients with more comorbidities or specific groups of illnesses/conditions (endocrine, mental illness or respiratory) had an increased risk of returning ED, so these groups may be prioritized in further research and health system initiatives to improve care before and after discharge.

## Introduction

After being discharged from hospital, patients experience a period of increased vulnerability that places them at increased risk of adverse events, known as “post-hospital syndrome” [1].

In order to respond to these increased needs, healthcare systems globally aim to ensure safe transitions of care when patients move from an acute care setting to home or to other provider [2]. Fragmented care after discharge has been associated with greater gaps in quality, more preventable hospitalizations, and decreased quality of life [3, 4]. Also, some initiatives to improve care coordination after discharge have decreased the risk of readmission, even if improvements are not consistent [5].

Inpatient care readmissions have been receiving considerable attention, mostly since their inclusion in pay-for-performance schemes in several countries [6]. Despite its relevance, these readmissions fail to capture the care of patients that visit emergency department (ED) after discharge and return home. Data on ED utilization shortly after discharge is comparatively scarce and mostly from US. Wadhera et al. identified 1,064,410 revisits (treat-and-discharge visit to ED, observation stay, and readmission) in the 30 days after 3,038,740 discharges of patients aged 65 years or over admitted for heart failure, acute myocardial infarction, or pneumonia [7]. The percentage of patients with at least one ED visit within 30 days of discharge varied between 21.7% (non-targeted conditions) and 26.4% (targeted conditions) in a study also aiming to study the effects of Medicare’s Hospital Readmissions Reduction Program [8]. Khera et al. described that nearly a third of all admissions received acute post-discharge care in observation units or the emergency department (heart failure: 30.7%; acute myocardial infarction: 26.9%; pneumonia: 24.8%) in 2008–16 [9]. A study at NHS hospitals in England, including chronic obstructive pulmonary disorder and heart failure patients, found that 1 in 5 returned to hospital within 30 days and that for nearly a quarter of ED visits there was no readmission [10].

In Portugal, health care provision is mainly ensured by a tax-funded National Health Service. A small flat rate is required to patients visiting ED, but around 60% of the population is exempted due to clinical and/or economic reasons and no previous referral is necessary. Due to its open-door policy, patients may see ED as a convenient way to access care, but its episodic nature may increase the number of providers caring for each patient and the risk of care fragmentation [11, 12]. Despite being a global concern, this problem has particular relevance in Portugal, since there is a high use of ED care. In 2011, there were over 70 ED visits per 100 population, while OECD (Organisation for Economic Co-operation and Development) countries average was 31 [13]. Additionally, 39% of triaged ED care visits in Portugal during 2019 had a low clinical priority [14]. Recent data indicate that only after the covid-19 pandemic there was a clear reduction in the volume of ED visits in Portugal [15].

Therefore, in this study we aimed to describe frequency and patient attributes associated with ED visits within 30 days of inpatient discharge in a Portuguese health region–Algarve.

## Methods

The study was approved by the hospital’s ethics committee (reference UIF 147/2018) and the data were irreversibly anonymized before being shared with researchers.

### Study design and context

We conducted a retrospective cohort study including adult patients discharged from a public hospital in Algarve, in 2016, and analysed their utilization of hospital ED and inpatient care within 30 days of discharge.

The Algarve health region is one of the five health regions in mainland Portugal, with 441,469 inhabitants in 2016 [16]. The region is served by one teaching public hospital comprising three hospital poles (Faro, Lagos and Portimão) with a capacity of 910 beds in 2016 and one specialized rehabilitation unit [17]. These acute hospital units provide non-elective outpatient (emergency department) and inpatient care (namely in intensive care units), as well as elective care (both inpatient and outpatient).

### Data sources and study population

We used data from inpatient and ED care, previously merged by the participating hospital using the National Health Service (NHS) number. The following records were excluded in this preliminary phase: discharges and ED visits without NHS number; patients transferred to other hospital or deceased; and patients transferred between ED units of studied hospital to avoid double counting. The data were then irreversibly anonymized and provided to the first author in a single database containing 27,432 inpatient admissions and the ED visits after discharge. The data on emergency care included date, mode of arrival and disposition for each ED visit. It also included the admission clinical priority for each ED visit, from Manchester Triage System. This triage system allocates the patient to the related urgency category (red, orange, yellow, green, and blue), with recommended waiting times ranging from “immediate” (red) to “four hours” (blue) [18]. For inpatient care, data included sex, age, admission type, principal and additional diagnoses (coded with ICD-9-CM), type of treatment (medical/surgical; from Diagnosis Related Groups), length of stay, and date of discharge for each admission. We added the Clinical Classifications Software [19] categorization and labels for diagnoses and considered in the study the first-level category (here referred to as “groups of illnesses/conditions”) and single-level diagnoses of principal diagnosis (here referred to as “illnesses/conditions”) and the number of comorbidities as the number of additional single-level diagnoses.

The study included all adult patients (aged ≥18 years) discharged during 2016 from the region’s public hospital inpatient departments. The period for ED visits included year 2016 and January 2017 to ensure follow up of patients discharged in December 2016. We excluded 5,684 admissions with age < 18 years and 4 admissions with inconsistent data.

### Recurrence to ED after discharge

The outcome of interest was having at least one ED visit within 30 days after discharge from hospital inpatient care. We also computed the number of ED visits in those 30 days and the number of days between discharge and the first ED visit.

### Statistical analysis

We described sample characteristics and calculated the 30-day ED admission rate after discharge for the total inpatient discharges and by characteristics of inpatient discharges. We characterized the number of days until the first admission to ED. We used the crude and age- and sex-adjusted odds ratios (OR) to analyse the association between the outcome (binary variable; 1: ED return) and selected variables. To choose the reference categories for groups of and individual illnesses/conditions, we looked for frequent categories with a relative frequency of outcome of interest similar to the studied sample.

For groups of individual illnesses, due to the high number of categories, the tables include only those statistically significant (p<0.05) and responsible for 90% of ED returns. A similar methodological option was adopted for illnesses and conditions, but with a 50% cut-off. Results for all categories are included in supporting information.

All analyses were run in IBM Statistical Package for Social Sciences (SPSS v24.0), with a level of significance of 5%.

## Results

### Studied population characteristics

The study included 21,744 adult admissions discharged in the Algarve health region in 2016 (Table 1). About 60% were female gender (59.66%), mean age was 58 years and patients aged 65 years or older accounted for over 45% of the admissions. Most admissions were urgent (81.75%). Diseases of the digestive system were a frequent cause of admission (11.35%), as well as respiratory diseases (10.49%). Injury and poisoning, neoplasms and diseases of the genitourinary system accounted for around 8–10% of admissions each. Admissions for mental or endocrine conditions were also relevant, but less frequent (4.07% and 2.17%). When analysing illnesses/conditions, some of the frequent were included in those groups, while some were not (e.g. congestive heart failure and hypertension, other complications of pregnancy and early or threatened labour). About half of admissions had two or less comorbidities (49.65%), while there were six or more comorbidities in 22.23% of cases. About one third of admissions received surgical treatment (32.22%). Median length of stay was 6 days (interquartile range: 3–12).

**Table 1:** Characteristics of included admissions.

	n (%)
**Total**	21744 (100)
**Sex**	
Male	8771 (40.34)
Female	12973 (59.66)
**Age (years)**	
18–35 (n,%)	4880 (22.44)
36–54 (n,%)	4385 (20.17)
55–64 (n,%)	2685 (12.35)
65–74 (n,%)	3274 (15.06)
≥75 (n,%)	6520 (29.99)
Average (±SD)	58 (22)
**Admission type**	
Non-urgent	3968 (18.25)
Urgent	17776 (81.75)
**Groups of illnesses/conditions**	
Diseases of the digestive system	2468 (11.35)
Diseases of the respiratory system	2280 (10.49)
Injury and poisoning	2110 (9.7)
Neoplasms	1943 (8.94)
Diseases of the genitourinary system	1783 (8.2)
Mental Illness	885 (4.07)
Endocrine nutritional and metabolic diseases and immunity disorders	471 (2.17)
**Illnesses/Conditions**	
Urinary tract infections	605 (2.78)
Congestive heart failure; nonhypertensive	434 (2)
Hypertension with complications and secondary hypertension	329 (1.51)
Other complications of pregnancy	291 (1.34)
Early or threatened labour	232 (1.07)
Mood disorders	232 (1.07)
Chronic obstructive pulmonary disease and bronchiectasis	214 (0.98)
Diabetes mellitus with complications	182 (0.84)
Fluid and electrolyte disorders	176 (0.81)
Other lower respiratory disease	152 (0.7)
Secondary malignancies	144 (0.66)
Cancer of rectum and anus	105 (0.48)
**Number of comorbidities**	
0	3256 (14.97)
1	4513 (20.76)
2	3027 (13.92)
3	2493 (11.47)
4	2075 (9.54)
5	1549 (7.12)
6	1258 (5.79)
7	1024 (4.71)
8	725 (3.33)
9	571 (2.63)
10	385 (1.77)
11	265 (1.22)
12	197 (0.91)
13	148 (0.68)
14 or over	258 (1.19)
**Treatment type**	
Surgical	7006 (32.22)
Medical	14738 (67.78)
**Length of stay (days)**	
0–3	7168 (32.97)
4–6	4435 (20.4)
7–11	4428 (20.36)
≥12	5713 (26.27)
Average (±SD)	10 (14)
Median (IQR)	6 (3–12)

Notes: IQR–interquartile range; SD–standard deviation.

For groups of illnesses/conditions, due to the high number of categories, the table includes only those responsible for 90% of ED returns and that were significantly associated with ED returns. Similarly for illnesses/ conditions, but the percentage was 50%. Results for all categories are included in supporting information.

### ED visits within 30 days of discharge

**Fig 1:**
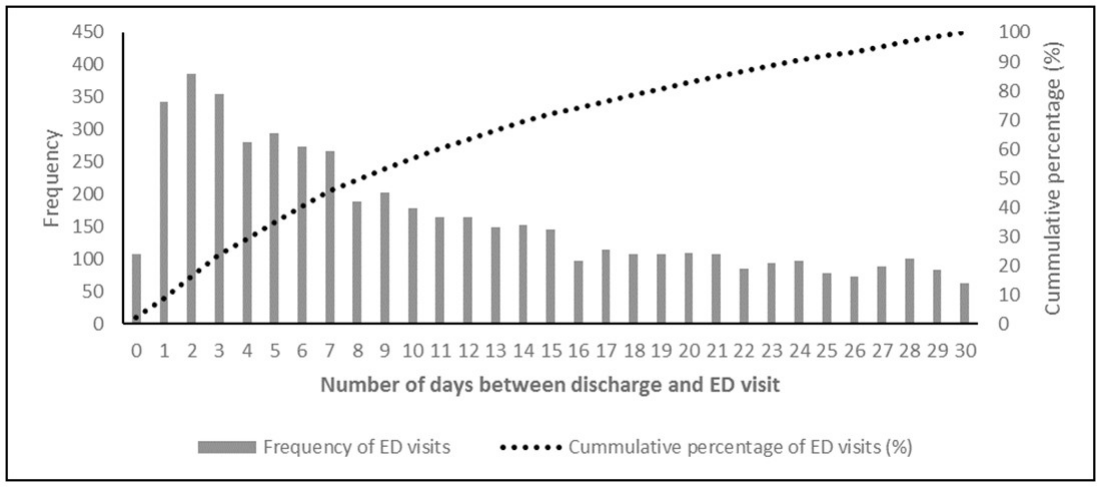
Distribution of ED visits per number of days between discharge and ED visit. Notes: ED–emergency department.

About a quarter of patients (23.26%, n = 5,058) visited ED at least once within 30 days of discharge. Average number of ED visits per patient in that period was 1.48, up to a maximum of 12 ED visits. The highest number of ED visits (n = 386, 7.63%) occurred in the second day after discharge (Fig 1). For about half of the patients that visited ED (53.28%), that occurred in the first nine days after discharge. A quarter visited in the first four days (29.08%) and three quarters in the first 17 days (76.33%).

After controlling for demographic characteristics (age and sex), some groups still presented increased odds of returning ED (Table 2). That was observed for patients who had been admitted urgently for inpatient care (25.12% returned; aOR: 1.823; 95% confidence interval: 1.657–2.006), compared to non-urgent. By group of illnesses/conditions, patients with endocrine conditions had a 56% higher risk of returning to ED (33.76%; aOR: 1.566; 1.256–1.951; reference group: neoplasms). Patients admitted for mental (27.12%; aOR: 1.421; 1.180–1.713) and respiratory illnesses had also an increased risk of ED return (32.02%; aOR: 1.308; 1.136–1.505). In contrast, for admissions for injury and poisoning (18.63%; aOR: 0.705; 0.604–0.822) and diseases of the digestive system (20.26%; aOR: 0.817; 0.706–0.945) we observed the opposite. The highest odds of ED return were observed by illness/condition, particularly for two obstetric conditions: early or threatened labour (58.62%; aOR: 6,844; 3,974–11,786) and other complications of pregnancy (33.33%; aOR: 2.428; 1.423–4.140). Some of other significant and relevant (aOR > 1.682) illnesses/conditions were included in groups of increased risk, such as respiratory diseases (other lower respiratory disease, chronic obstructive pulmonary disease–COPD), endocrine diseases (diabetes mellitus with complications), or mental illness (mood disorders). However, some conditions outside those groups also emerged and had increased odds for returning to ED: fluid and electrolyte disorders, secondary malignancies, hypertension with complications and secondary hypertension, urinary tract infections and congestive heart failure. The adjusted odds-ratios for all groups of and individual conditions/illnesses are included in supporting information. The risk of ED return tended to increase as the number of comorbidities increased (maximum: 14 or over comorbidities; 40.7%, aOR: 2.465, 1.885–3.223). Medical admissions had 50% higher odds of returning to ED, compared to surgical admissions (25.93%, aOR: 1.532, 1.425–1.648). Longer lengths of stay were associated with returning to ED (maximum: 12 or more days; 29.84%, aOR: 1.729, 1.577–1.896).

**Table 2:** Frequency of ED visits within 30 days after hospital discharge and age- and sex- adjusted odds-ratios.

	ED visits (n, % admissions)	Age- and sex-adjusted OR
		(95% CI, p)
**Total**	5058 (23.26)	-
**Sex**		
Male	2231 (25.44)	1.18 (1.103–1.262); p<0.001
Female	2827 (21.79)	Ref.
**Age (years)**		
18–35	962 (19.71)	Ref.
36–54	825 (18.81)	0.906 (0.816–1.006); p = 0.065
55–64	531 (19.78)	0.932 (0.825–1.053); p = 0.259
65–74	747 (22.82)	1.123 (1.004–1.255); p = 0.042
≥75	1993 (30.57)	1.704 (1.557–1.866); p<0.001
**Admission type**		
Non-urgent	593 (14.94)	Ref.
Urgent	4465 (25.12)	1.823 (1.657–2.006); p<0.001
**Groups of illnesses/conditions**		
Diseases of the digestive system	500 (20.26)	0.817 (0.706–0.945); p = 0.007
Diseases of the respiratory system	730 (32.02)	1.308 (1.136–1.505); p<0.001
Injury and poisoning	393 (18.63)	0.705 (0.604–0.822); p<0.001
Neoplasms	449 (23.11)	Ref.
Diseases of the genitourinary system	482 (27.03)	1.163 (1.001–1.352); p = 0.048
Mental Illness	240 (27.12)	1.421 (1.18–1.713); p<0.001
Endocrine nutritional and metabolic diseases and immunity disorders	159 (33.76)	1.566 (1.256–1.951); p<0.001
**Illnesses/Conditions**		
Urinary tract infections	210 (34.71)	1.691 (1.038–2.757); p = 0.035
Congestive heart failure; nonhypertensiv0065	155 (35.71)	1.682 (1.021–2.77); p = 0.041
Hypertension with complications and secondary hypertension	118 (35.87)	1.708 (1.024–2.848); p = 0.04
Other complications of pregnancy	97 (33.33)	2.428 (1.423–4.14); p = 0.001
Early or threatened labour	136 (58.62)	6.844 (3.974–11.786); p<0.001
Mood disorders	64 (27.59)	1.754 (1.017–3.025); p = 0.043
Chronic obstructive pulmonary disease and bronchiectasis	73 (34.11)	1.721 (1.004–2.952); p = 0.048
Diabetes mellitus with complications	60 (32.97)	1.811 (1.041–3.151); p = 0.036
Fluid and electrolyte disorders	75 (42.61)	2.231 (1.289–3.86); p = 0.004
Other lower respiratory disease	56 (36.84)	1.881 (1.068–3.311); p = 0.029
Secondary malignancies	49 (34.03)	1.983 (1.116–3.525); p = 0.02
Cancer of rectum and anus	24 (22.86)	Ref.
**Number of comorbidities**		
0	631 (19.38)	Ref.
1	756 (16.75)	0.833 (0.74–0.937); p = 0.002
2	600 (19.82)	1.008 (0.889–1.142); p = 0.902
3	545 (21.86)	1.113 (0.978–1.268); p = 0.105
4	474 (22.84)	1.153 (1.006–1.322); p = 0.041
5	418 (26.99)	1.411 (1.22–1.632); p<0.001
6	372 (29.57)	1.58 (1.355–1.842); p<0.001
7	320 (31.25)	1.692 (1.437–1.992); p<0.001
8	239 (32.97)	1.769 (1.473–2.124); p<0.001
9	204 (35.73)	2.003 (1.645–2.44); p<0.001
10	151 (39.22)	2.329 (1.856–2.922); p<0.001
11	99 (37.36)	2.14 (1.636–2.798); p<0.001
12	82 (41.62)	2.508 (1.856–3.388); p<0.001
13	62 (41.89)	2.589 (1.839–3.645); p<0.001
14 or over	105 (40.7)	2.465 (1.885–3.223); p<0.001
**Treatment type**		
Surgical	1236 (17.64)	Ref.
Medical	3822 (25.93)	1.532 (1.425–1.648); p<0.001
**Length of stay (days)**		
0–3	1251 (17.45)	Ref.
4–6	919 (20.72)	1.204 (1.094–1.325); p<0.001
7–11	1183 (26.72)	1.546 (1.405–1.702); p<0.001
≥12	1705 (29.84)	1.729 (1.577–1.896); p<0.001

Notes: CI–confidence interval. ED–emergency department. OR–odds ratio. Ref.–reference group.

Odds ratios were computed for each variable individually and adjusted for age and sex.

For groups of illnesses/conditions, due to the high number of categories, the table includes only those responsible for 90% of ED returns and that were significantly associated with ED returns. Similarly for illnesses/ conditions, but the percentage was 50%. Results for all categories are included in supporting information.

About 75% of patients were triaged yellow (45.20%), orange (29.64%), or red (1.11%) when visiting ED after inpatient care discharge (Table 3). Readmitted patients accounted for 28.27% of those that visited ED within 30 days of discharge. For the remaining, 29.66% had record of ambulatory follow-up (hospital or primary care) and 42.07% left ED without mention of planned follow up.

**Table 3:** Characteristics of ED visits within 30 days after discharge.

	N (%)
**Total ED visits**	5,058 (100.00)
**Admission clinical priority**	
Red	56 (1.11)
Orange	1,499 (29.64)
Yellow	2,286 (45.20)
Green	665 (13.15)
Blue	6 (0.12)
White or Non-triaged	546 (10.79)
**Disposition**	
No follow-up planned	2,128 (42.07)
Admit to inpatient care	1,430 (28.27)
Primary care for follow-up	788 (15.58)
Hospital ambulatory care follow up	430 (8.50)
Left prior to completing visit	183 (3.62)
Death	85 (1.68)
Other dispositions	14 (0.28)

Notes: ED–emergency department.

## Discussion

This study showed that nearly a quarter of discharged patients returned to ED in the first month after admission, which is consistent with earlier studies [7, 9, 10].

As expected, our results showed that the risk for returning ED was higher for patients with urgent admission, more comorbidities, or longer inpatient stays, who probably have poorer health. In a country with a disproportionately high utilization of ED care [13], there are some concerns about the appropriateness of ED utilization. However, our results indicate that these patients returned ED for severe reasons, since the percentage of those triaged green, blue and white (24.06%) was lower than for aggregate ED use (37.23% in 2016) [17]. Also, 28.27% of ED visits ended with an inpatient admission, which is higher than observed overall (6.69% in 2016) [17]. Future studies may also consider the outcome of inpatient care (if the patient deceased in hospital or after) and provide evidence on how the phase of illness trajectory influences ED returns and the specific needs of people living with palliative conditions.

These returns to ED care may share some underlying reasons with those described for readmissions, from an early discharge to inadequate transitions of care [20], changes in medication or complex regimes [21], the “post-hospital syndrome” [1] and be influenced by health literacy [22]. Therefore, changes to healthcare delivery and organization to achieve those improvements call for integrated actions from several providers, namely acute and primary care. The regular monitoring of ED returns by hospitals and primary care providers would be a relatively low-resource initiative to raise awareness. These data would also be valuable to guide the design of interventions to improve transitions of care from inpatient to outpatient care. The design phase is critical, since some of these interventions do not achieve the expected results and more intensive and/or longer interventions may be necessary, what requires a careful analysis of expected cost-effectiveness [20]. Our results also highlight the importance of the period before and shortly after discharge, since half of ED returns occurred within 9 days of discharge. Moreover, it is of concern that records indicate that over 40% of patients returning ED left hospital without mention of a planned follow up.

We observed that, for some illnesses/conditions, the risk of ED visits was higher. Specific obstetric illnesses/conditions had particularly higher rates. In Portugal, it is frequent that the admission for delivery begins with an ED visit, so these results warrant further study, aiming to differentiate ED visits that are expected. Respiratory illnesses/conditions as a whole, and chronic obstructive pulmonary disease and other lower respiratory disease specifically, were also relevant. Previous studies have shown that telemonitoring after discharge has reduced hospital utilization for COPD patients [23] and there are financial incentives for hospitals to implement it [24]. Patients admitted for mental illnesses/conditions are also a group of interest for possible improvements in healthcare, since these patients have a higher risk of becoming ED frequent users [25]. Community based multidisciplinary and multi-level teams endowed with active care plan management are interventions that promote both effectiveness and adequacy of care and prevent ED recurrence [26]. Despite the importance of specific illnesses/conditions, interventions to reduce avoidable ED returns should take into account the frequency of multimorbidity. In our study, more than half of patients had at least three comorbidities and the risk of ED returns rose as the number of comorbidities increased.

One of the limitations of our study is that we did not analyse the clinical reason for visiting ED. The availability of these data, combined with the perspective of patients, would provide a deeper understanding of why patients return ED. The inclusion of one year at a specific region is also a limitation; however, the volume of cases allowed us to conduct robust analyses. Despite that, future studies may consider the effect of demographic and clinical characteristics (principal illness/condition and comorbidities) together and identify relevant comorbidities. It was not possible to study the association between ED returns and relevant patients’ characteristics, such as education or income, which could have allowed us identifying additional attributes associated with ED returns. The utilization of other sources of care (hospital and primary care) during the post-discharge period was not considered. That would allow us to assess if visiting ED was linked with lack of access to scheduled care. The frequency of our outcome of interest may be underestimated, since ED visits in private hospitals were not possible to include. Finally, completeness of coding may have influenced our results, but its impact may be limited by the fact that it may be homogeneously distributed across patient groups.

## Conclusions

In summary, our study showed that patients return ED after inpatient discharge frequently and readmissions capture a limited share of that utilization. Having in mind that patients return ED for severe reasons, future research is needed on the avoidable share of this utilization. Patients with more comorbidities or specific groups of illnesses/conditions (endocrine, respiratory or mental illness) had an increased risk of returning ED, so these groups may be prioritized in further research and health system initiatives to improve care before and after discharge.

## Supporting information

S1 Table Frequency of ED visits within 30 days after hospital discharge and age- and sex- adjusted odds-ratios (groups of illnesses/conditions). (DOCX)

S2 Table Frequency of ED visits within 30 days after hospital discharge and age- and sex- adjusted odds-ratios (illnesses/conditions). (DOCX)
