# Supplementary material for: Frequency and patient attributes associated with emergency department visits after discharge: Retrospective cohort study
Source: PLoS One. 2022 Oct 14;17(10):e0275215. doi: 10.1371/journal.pone.0275215 (PMC9565411; doi:10.1371/journal.pone.0275215)
Supplement: S1 Table — (DOCX) [file pone.0275215.s001.docx]

**S1 Table – Frequency of ED visits within 30 days after hospital discharge and age- and sex- adjusted odds-ratios (groups of illnesses/conditions)**

|  | **ED Visits (n, % admissions)** | | **Age- and Sex-**  **Adjusted Odds Ratio** | | | |
| --- | --- | --- | --- | --- | --- | --- |
|  |  |  | **OR** | **Min** | **Max** | **ρ** |
| **Total** | 5,058 | 23.26 | - | | | |
| **Sex** |  |  |  |  |  |  |
| Male | 2,231 | 25.44 | 1.1767 | 1.0956 | 1.2638 | 0.0000 |
| Female | 2,827 | 21.79 | Ref. | | | |
| **Age** |  |  |  |  |  |  |
| 18-35 | 962 | 19.71 | Ref. | | | |
| 36-54 | 825 | 18.81 | 0.9337 | 0.8275 | 1.0536 | 0.2660 |
| 55-64 | 531 | 19.78 | 0.9908 | 0.8542 | 1.1494 | 0.9030 |
| 65-74 | 747 | 22.82 | 1.2095 | 1.0499 | 1.3933 | 0.0080 |
| ≥75 | 1,993 | 30.57 | 1.7736 | 1.5608 | 2.0155 | 0.0000 |
| **Groups of illnesses/conditions** |  |  |  |  |  |  |
| Infectious and parasitic diseases | 82 | 28.57 | 1.3035 | 0.9854 | 1.7243 | 0.0630 |
| Neoplasms | 449 | 23.11 | Ref. | | | |
| Endocrine nutritional and metabolic diseases and immunity disorders | 159 | 33.76 | 1.5656 | 1.2560 | 1.9515 | 0.0000 |
| Diseases of the blood and blood-forming organs | 45 | 26.32 | 1.0950 | 0.7647 | 1.5682 | 0.6200 |
| Mental Illness | 240 | 27.12 | 1.4214 | 1.1796 | 1.7127 | 0.0000 |
| Diseases of the nervous system and sense organs | 75 | 15.31 | 0.6545 | 0.4999 | 0.8570 | 0.0020 |
| Diseases of the circulatory system | 763 | 25.00 | 0.9456 | 0.8253 | 1.0834 | 0.4200 |
| Diseases of the respiratory system | 730 | 32.02 | 1.3077 | 1.1363 | 1.5049 | 0.0000 |
| Diseases of the digestive system | 500 | 20.26 | 0.8171 | 0.7064 | 0.9453 | 0.0070 |
| Diseases of the genitourinary system | 482 | 27.03 | 1.1635 | 1.0011 | 1.3521 | 0.0480 |
| Complications of pregnancy childbirth and the puerperium | 856 | 19.42 | 1.0637 | 0.9031 | 1.2529 | 0.4600 |
| Diseases of the skin and subcutaneous tissue | 56 | 25.81 | 1.1649 | 0.8422 | 1.6113 | 0.3560 |
| Diseases of the musculoskeletal system and connective tissue | 69 | 14.47 | 0.5674 | 0.4299 | 0.7489 | 0.0000 |
| Congenital anomalies | 4 | 16.00 | 0.7420 | 0.2526 | 2.1791 | 0.5870 |
| Injury and poisoning | 393 | 18.63 | 0.7048 | 0.6042 | 0.8220 | 0.0000 |
| Symptoms signs and ill-defined conditions and factors influencing health status | 135 | 22.20 | 0.8637 | 0.6928 | 1.0768 | 0.1930 |
| Residual codes unclassified and all E codes | 20 | 28.99 | 1.4825 | 0.8682 | 2.5313 | 0.1490 |

Notes: ED – emergency department. Min – Minimum (lower limit of 95% confidence interval). Max – Maximum (upper limit of 95% confidence interval). OR – odds ratio. Ref. – reference group.
