# Supplementary material for: Frequency and patient attributes associated with emergency department visits after discharge: Retrospective cohort study
Source: PLoS One. 2022 Oct 14;17(10):e0275215. doi: 10.1371/journal.pone.0275215 (PMC9565411; doi:10.1371/journal.pone.0275215)
Supplement: S2 Table — (DOCX) [file pone.0275215.s002.docx]

**S2 Table – Frequency of ED visits within 30 days after hospital discharge and age- and sex- adjusted odds-ratios (illnesses/conditions)**

|  | **ED Visits (n, % of admissions)** | | **Age- and Sex- Adjusted Odds Ratio** | | | |
| --- | --- | --- | --- | --- | --- | --- |
|  |  |  | **OR** | **Min** | **Max** | **ρ** |
| **Total** | 5,058 | 23.26% | - | | | |
| **Sex** |  |  |  |  |  |  |
| Male | 2,231 | 25.44% | 1.1594 | 1.0730 | 1.2527 | 0.0000 |
| Female | 2,827 | 21.79% | 1.0000 |  |  |  |
| **Age** |  |  |  |  |  |  |
| 18-35 | 962 | 19.71% | 1.0000 |  |  |  |
| 36-54 | 825 | 18.81% | 0.9426 | 0.8295 | 1.0711 | 0.3640 |
| 55-64 | 531 | 19.78% | 1.0048 | 0.8568 | 1.1784 | 0.9530 |
| 65-74 | 747 | 22.82% | 1.2035 | 1.0315 | 1.4042 | 0.0190 |
| ≥75 | 1,993 | 30.57% | 1.6953 | 1.4675 | 1.9584 | 0.0000 |
| **Ilnesses/Conditions** |  |  |  |  |  |  |
| Tuberculosis | <5 |  |  |  |  |  |
| Septicemia (except in labor) | 41 | 35.34% | 1.7775 | 0.9780 | 3.2305 | 0.0590 |
| Bacterial infection; unspecified site | <5 |  |  |  |  |  |
| Mycoses | <5 |  |  |  |  |  |
| HIV infection | 14 | 28.57% | 1.7317 | 0.7980 | 3.7578 | 0.1650 |
| Hepatitis | 10 | 43.48% | 3.0655 | 1.1884 | 7.9073 | 0.0210 |
| Viral infection | 7 | 36.84% | 2.1649 | 0.7625 | 6.1465 | 0.1470 |
| Other infections; including parasitic | <5 |  |  |  |  |  |
| Sexually transmitted infections (not HIV or hepatitis) | <5 |  |  |  |  |  |
| Cancer of head and neck | 15 | 28.85% | 1.6245 | 0.7618 | 3.4641 | 0.2090 |
| Cancer of esophagus | <5 |  |  |  |  |  |
| Cancer of stomach | 21 | 33.33% | 1.4993 | 0.7460 | 3.0135 | 0.2550 |
| Cancer of colon | 45 | 30.00% | 1.4155 | 0.7948 | 2.5210 | 0.2380 |
| Cancer of rectum and anus | 24 | 22.86% | Ref. | | | |
| Cancer of liver and intrahepatic bile duct | <5 |  |  |  |  |  |
| Cancer of pancreas | 16 | 61.54% | 5.4231 | 2.1661 | 13.5778 | 0.0000 |
| Cancer of other GI organs; peritoneum | 9 | 39.13% | 2.0378 | 0.7815 | 5.3141 | 0.1450 |
| Cancer of bronchus; lung | 38 | 48.10% | 3.1668 | 1.6724 | 5.9965 | 0.0000 |
| Other non-epithelial cancer of skin | 7 | 36.84% | 1.8155 | 0.6391 | 5.1569 | 0.2630 |
| Cancer of breast | 23 | 8.13% | 0.3729 | 0.1988 | 0.6994 | 0.0020 |
| Cancer of uterus | 10 | 18.52% | 0.9084 | 0.3965 | 2.0813 | 0.8200 |
| Cancer of cervix | 10 | 29.41% | 1.9311 | 0.8055 | 4.6297 | 0.1400 |
| Cancer of ovary | 10 | 29.41% | 1.7356 | 0.7249 | 4.1552 | 0.2160 |
| Cancer of other female genital organs | <5 |  |  |  |  |  |
| Cancer of prostate | 10 | 15.38% | 0.5944 | 0.2626 | 1.3451 | 0.2120 |
| Cancer of testis | <5 |  |  |  |  |  |
| Cancer of other male genital organs | <5 |  |  |  |  |  |
| Cancer of bladder | 37 | 17.70% | 0.6886 | 0.3854 | 1.2305 | 0.2080 |
| Cancer of kidney and renal pelvis | 10 | 22.22% | 1.0467 | 0.4508 | 2.4299 | 0.9150 |
| Cancer of other urinary organs | <5 |  |  |  |  |  |
| Cancer of brain and nervous system | 11 | 29.73% | 1.7016 | 0.7316 | 3.9578 | 0.2170 |
| Cancer of thyroid | <5 |  |  |  |  |  |
| Hodgkins disease | <5 |  |  |  |  |  |
| Non-Hodgkins lymphoma | 8 | 30.77% | 1.6138 | 0.6211 | 4.1933 | 0.3260 |
| Leukemias | <5 |  |  |  |  |  |
| Multiple myeloma | <5 |  |  |  |  |  |
| Cancer; other and unspecified primary | <5 |  |  |  |  |  |
| Secondary malignancies | 49 | 34.03% | 1.9834 | 1.1161 | 3.5245 | 0.0200 |
| Malignant neoplasm without specification of site | <5 |  |  |  |  |  |
| Neoplasms of unspecified nature or uncertain behavior | 13 | 19.70% | 0.9230 | 0.4303 | 1.9798 | 0.8370 |
| Maintenance chemotherapy; radiotherapy | 5 | 20.00% | 1.0568 | 0.3575 | 3.1238 | 0.9200 |
| Benign neoplasm of uterus | 28 | 23.14% | 1.5149 | 0.8058 | 2.8479 | 0.1970 |
| Other and unspecified benign neoplasm | 26 | 15.29% | 0.7090 | 0.3807 | 1.3202 | 0.2780 |
| Thyroid disorders | 12 | 18.46% | 0.9224 | 0.4231 | 2.0110 | 0.8390 |
| Diabetes mellitus with complications | 60 | 32.97% | 1.8110 | 1.0409 | 3.1509 | 0.0360 |
| Other endocrine disorders | <5 |  |  |  |  |  |
| Nutritional deficiencies | <5 |  |  |  |  |  |
| Gout and other crystal arthropathies | <5 |  |  |  |  |  |
| Fluid and electrolyte disorders | 75 | 42.61% | 2.2307 | 1.2891 | 3.8601 | 0.0040 |
| Other nutritional; endocrine; and metabolic disorders | 5 | 21.74% | 1.1930 | 0.3986 | 3.5707 | 0.7520 |
| Deficiency and other anemia | 23 | 29.11% | 1.3131 | 0.6721 | 2.5655 | 0.4250 |
| Coagulation and hemorrhagic disorders | 10 | 41.67% | 2.5881 | 1.0142 | 6.6043 | 0.0470 |
| Diseases of white blood cells | 12 | 21.43% | 1.0138 | 0.4611 | 2.2293 | 0.9730 |
| Encephalitis (except that caused by tuberculosis or sexually transmitted disease) | <5 |  |  |  |  |  |
| Other CNS infection and poliomyelitis | <5 |  |  |  |  |  |
| Multiple sclerosis | <5 |  |  |  |  |  |
| Other hereditary and degenerative nervous system conditions | 8 | 22.22% | 1.0567 | 0.4239 | 2.6343 | 0.9060 |
| Epilepsy; convulsions | 16 | 21.05% | 0.9839 | 0.4792 | 2.0202 | 0.9650 |
| Headache; including migraine | 5 | 31.25% | 2.0749 | 0.6521 | 6.6018 | 0.2160 |
| Retinal detachments; defects; vascular occlusion; and retinopathy | <5 |  |  |  |  |  |
| Inflammation; infection of eye (except that caused by tuberculosis or sexually transmitteddi..) | 5 | 21.74% | 0.9845 | 0.3281 | 2.9540 | 0.9780 |
| Other eye disorders | 5 | 23.81% | 1.0728 | 0.3535 | 3.2561 | 0.9010 |
| Otitis media and related conditions | <5 |  |  |  |  |  |
| Conditions associated with dizziness or vertigo | <5 |  |  |  |  |  |
| Other ear and sense organ disorders | 5 | 9.62% | 0.4505 | 0.1606 | 1.2638 | 0.1300 |
| Other nervous system disorders | 18 | 15.25% | 0.7634 | 0.3861 | 1.5093 | 0.4380 |
| Heart valve disorders | 7 | 36.84% | 1.8803 | 0.6623 | 5.3384 | 0.2360 |
| Peri-; endo-; and myocarditis; cardiomyopathy (except that caused by tuberculosis or sexuall..) | 17 | 24.64% | 1.2981 | 0.6336 | 2.6593 | 0.4760 |
| Essential hypertension | <5 |  |  |  |  |  |
| Hypertension with complications and secondary hypertension | 118 | 35.87% | 1.7078 | 1.0241 | 2.8480 | 0.0400 |
| Acute myocardial infarction | 104 | 25.06% | 1.1385 | 0.6841 | 1.8949 | 0.6180 |
| Coronary atherosclerosis and other heart disease | <5 |  |  |  |  |  |
| Nonspecific chest pain | <5 |  |  |  |  |  |
| Pulmonary heart disease | 33 | 29.73% | 1.4051 | 0.7601 | 2.5975 | 0.2780 |
| Other and ill-defined heart disease | <5 |  |  |  |  |  |
| Conduction disorders | 37 | 21.14% | 0.7632 | 0.4249 | 1.3707 | 0.3660 |
| Cardiac dysrhythmias | 38 | 25.17% | 1.0576 | 0.5872 | 1.9047 | 0.8520 |
| Congestive heart failure; nonhypertensive | 155 | 35.71% | 1.6819 | 1.0211 | 2.7704 | 0.0410 |
| Acute cerebrovascular disease | 165 | 18.88% | 0.7380 | 0.4528 | 1.2029 | 0.2230 |
| Occlusion or stenosis of precerebral arteries | <5 |  |  |  |  |  |
| Transient cerebral ischemia | 25 | 20.33% | 0.8684 | 0.4598 | 1.6400 | 0.6640 |
| Peripheral and visceral atherosclerosis | 17 | 30.91% | 1.3739 | 0.6584 | 2.8668 | 0.3970 |
| Aortic and peripheral arterial embolism or thrombosis | <5 |  |  |  |  |  |
| Phlebitis; thrombophlebitis and thromboembolism | 19 | 25.33% | 1.2473 | 0.6216 | 2.5029 | 0.5340 |
| Varicose veins of lower extremity | 5 | 7.58% | 0.3276 | 0.1179 | 0.9104 | 0.0320 |
| Hemorrhoids | 7 | 21.88% | 1.2577 | 0.4824 | 3.2788 | 0.6390 |
| ther diseases of veins and lymphatics | <5 |  |  |  |  |  |
| Pneumonia (except that caused by tuberculosis or sexually transmitted disease) | 357 | 32.87% | 1.4791 | 0.9190 | 2.3808 | 0.1070 |
| Acute and chronic tonsillitis | 8 | 36.36% | 2.5787 | 0.9604 | 6.9238 | 0.0600 |
| Acute bronchitis | 125 | 34.25% | 1.5279 | 0.9197 | 2.5385 | 0.1020 |
| Other upper respiratory infections | <5 |  |  |  |  |  |
| Chronic obstructive pulmonary disease and bronchiectasis | 73 | 34.11% | 1.7215 | 1.0041 | 2.9516 | 0.0480 |
| Asthma | 28 | 27.72% | 1.4513 | 0.7691 | 2.7385 | 0.2500 |
| Aspiration pneumonitis; food/vomitus | 27 | 49.09% | 2.7353 | 1.3555 | 5.5197 | 0.0050 |
| Pleurisy; pneumothorax; pulmonary collapse | 20 | 25.32% | 1.2692 | 0.6388 | 2.5216 | 0.4960 |
| Respiratory failure; insufficiency; arrest (adult) | 15 | 27.27% | 1.2085 | 0.5695 | 2.5646 | 0.6220 |
| Lung disease due to external agents | <5 |  |  |  |  |  |
| Other lower respiratory disease | 56 | 36.84% | 1.8807 | 1.0682 | 3.3111 | 0.0290 |
| Other upper respiratory disease | 14 | 14.29% | 0.6835 | 0.3292 | 1.4190 | 0.3070 |
| Intestinal infection | 9 | 18.37% | 0.7423 | 0.3144 | 1.7524 | 0.4970 |
| Disorders of teeth and jaw | <5 |  |  |  |  |  |
| Diseases of mouth; excluding dental | <5 |  |  |  |  |  |
| Esophageal disorders | 5 | 17.86% | 0.7374 | 0.2519 | 2.1586 | 0.5780 |
| Gastroduodenal ulcer (except hemorrhage) | 7 | 21.21% | 0.9417 | 0.3620 | 2.4498 | 0.9020 |
| Gastritis and duodenitis | <5 |  |  |  |  |  |
| Other disorders of stomach and duodenum | 11 | 45.83% | 2.7839 | 1.0964 | 7.0685 | 0.0310 |
| Appendicitis and other appendiceal conditions | 32 | 15.24% | 0.7787 | 0.4278 | 1.4175 | 0.4130 |
| Abdominal hernia | 61 | 16.58% | 0.7013 | 0.4108 | 1.1971 | 0.1930 |
| Regional enteritis and ulcerative colitis | 11 | 27.50% | 1.6404 | 0.7106 | 3.7869 | 0.2460 |
| Intestinal obstruction without hernia | 38 | 34.55% | 1.8523 | 1.0113 | 3.3927 | 0.0460 |
| Diverticulosis and diverticulitis | 24 | 24.49% | 1.1033 | 0.5751 | 2.1167 | 0.7670 |
| Anal and rectal conditions | 24 | 25.81% | 1.4014 | 0.7278 | 2.6985 | 0.3130 |
| Peritonitis and intestinal abscess | 10 | 29.41% | 1.5193 | 0.6353 | 3.6333 | 0.3470 |
| Biliary tract disease | 146 | 17.10% | 0.7532 | 0.4604 | 1.2323 | 0.2590 |
| Other liver diseases | 22 | 40.74% | 2.5329 | 1.2417 | 5.1665 | 0.0110 |
| Pancreatic disorders (not diabetes) | 29 | 18.95% | 0.8275 | 0.4484 | 1.5272 | 0.5450 |
| Gastrointestinal hemorrhage | 39 | 24.68% | 1.0755 | 0.5992 | 1.9305 | 0.8070 |
| Noninfectious gastroenteritis | 10 | 27.03% | 1.2089 | 0.5104 | 2.8637 | 0.6660 |
| Other gastrointestinal disorders | 18 | 19.57% | 0.8180 | 0.4099 | 1.6327 | 0.5690 |
| Nephritis; nephrosis; renal sclerosis | <5 |  |  |  |  |  |
| Acute and unspecified renal failure | 9 | 33.33% | 1.5489 | 0.6138 | 3.9086 | 0.3540 |
| Chronic renal failure | 47 | 28.83% | 1.3162 | 0.7436 | 2.3299 | 0.3460 |
| Urinary tract infections | 210 | 34.71% | 1.6915 | 1.0376 | 2.7574 | 0.0350 |
| Calculus of urinary tract | 35 | 21.60% | 1.1217 | 0.6194 | 2.0313 | 0.7050 |
| Other diseases of kidney and ureters | 32 | 38.55% | 2.2891 | 1.2080 | 4.3378 | 0.0110 |
| Other diseases of bladder and urethra | 10 | 25.00% | 1.1211 | 0.4778 | 2.6306 | 0.7930 |
| Genitourinary symptoms and ill-defined conditions | 29 | 28.16% | 1.2212 | 0.6505 | 2.2929 | 0.5340 |
| Hyperplasia of prostate | 22 | 22.68% | 0.8842 | 0.4561 | 1.7140 | 0.7160 |
| Inflammatory conditions of male genital organs | <5 |  |  |  |  |  |
| Nonmalignant breast conditions | <5 |  |  |  |  |  |
| Inflammatory diseases of female pelvic organs | 14 | 20.29% | 1.2365 | 0.5834 | 2.6210 | 0.5800 |
| Endometriosis | <5 |  |  |  |  |  |
| Prolapse of female genital organs | 19 | 24.36% | 1.2741 | 0.6364 | 2.5508 | 0.4940 |
| Menstrual disorders | 8 | 27.59% | 1.9311 | 0.7541 | 4.9447 | 0.1700 |
| Ovarian cyst | 14 | 24.56% | 1.5581 | 0.7260 | 3.3441 | 0.2550 |
| Menopausal disorders | <5 |  |  |  |  |  |
| Female infertility | <5 |  |  |  |  |  |
| Other female genital disorders | 19 | 15.08% | 0.7990 | 0.4074 | 1.5672 | 0.5140 |
| Contraceptive and procreative management | <5 |  |  |  |  |  |
| Spontaneous abortion | 32 | 24.06% | 1.5498 | 0.8355 | 2.8746 | 0.1650 |
| Induced abortion | 20 | 20.62% | 1.2743 | 0.6444 | 2.5199 | 0.4860 |
| Ectopic pregnancy | 18 | 35.29% | 2.6657 | 1.2666 | 5.6099 | 0.0100 |
| Other complications of pregnancy | 97 | 33.33% | 2.4276 | 1.4234 | 4.1402 | 0.0010 |
| Hemorrhage during pregnancy; abruptio placenta; placenta previa | 31 | 40.26% | 3.2906 | 1.7061 | 6.3466 | 0.0000 |
| Hypertension complicating pregnancy; childbirth and the puerperium | 21 | 25.61% | 1.6814 | 0.8472 | 3.3371 | 0.1370 |
| Early or threatened labor | 136 | 58.62% | 6.8439 | 3.9740 | 11.7864 | 0.0000 |
| Prolonged pregnancy | 14 | 16.09% | 0.9269 | 0.4403 | 1.9513 | 0.8420 |
| Diabetes or abnormal glucose tolerance complicating pregnancy; childbirth; or the puerperium | 20 | 21.05% | 1.2992 | 0.6555 | 2.5749 | 0.4530 |
| Malposition; malpresentation | 28 | 13.02% | 0.7280 | 0.3921 | 1.3517 | 0.3150 |
| Fetopelvic disproportion; obstruction | 102 | 16.86% | 0.9846 | 0.5851 | 1.6569 | 0.9530 |
| Previous C-section | 6 | 15.79% | 0.9191 | 0.3409 | 2.4778 | 0.8680 |
| Fetal distress and abnormal forces of labor | 19 | 15.97% | 0.9273 | 0.4690 | 1.8336 | 0.8280 |
| Polyhydramnios and other problems of amniotic cavity | 43 | 15.64% | 0.8987 | 0.5051 | 1.5989 | 0.7160 |
| Umbilical cord complication | 7 | 16.67% | 0.9675 | 0.3776 | 2.4792 | 0.9450 |
| OB-related trauma to perineum and vulva | 63 | 11.41% | 0.6270 | 0.3646 | 1.0780 | 0.0910 |
| Forceps delivery | <5 |  |  |  |  |  |
| Other complications of birth; puerperium affecting management of mother | 82 | 22.78% | 1.4313 | 0.8381 | 2.4444 | 0.1890 |
| Other pregnancy and delivery including normal | 113 | 11.10% | 0.6044 | 0.3612 | 1.0111 | 0.0550 |
| Skin and subcutaneous tissue infections | 41 | 23.84% | 1.1863 | 0.6652 | 2.1159 | 0.5630 |
| Other inflammatory condition of skin | 8 | 47.06% | 3.1112 | 1.0720 | 9.0293 | 0.0370 |
| Chronic ulcer of skin | 6 | 37.50% | 2.0853 | 0.6833 | 6.3635 | 0.1970 |
| Other skin disorders | <5 |  |  |  |  |  |
| Infective arthritis and osteomyelitis (except that caused by tuberculosis or sexually transm..) | 5 | 20.83% | 0.8508 | 0.2859 | 2.5320 | 0.7720 |
| Rheumatoid arthritis and related disease | <5 |  |  |  |  |  |
| Osteoarthritis | 18 | 10.17% | 0.4030 | 0.2062 | 0.7874 | 0.0080 |
| Other non-traumatic joint disorders | 5 | 20.83% | 1.0057 | 0.3383 | 2.9893 | 0.9920 |
| Spondylosis; intervertebral disc disorders; other back problems | 15 | 13.76% | 0.6393 | 0.3131 | 1.3057 | 0.2200 |
| Pathological fracture | 5 | 38.46% | 2.3503 | 0.6985 | 7.9081 | 0.1670 |
| Acquired foot deformities | <5 |  |  |  |  |  |
| Other acquired deformities | <5 |  |  |  |  |  |
| Systemic lupus erythematosus and connective tissue disorders | <5 |  |  |  |  |  |
| Other connective tissue disease | 9 | 16.67% | 0.7455 | 0.3180 | 1.7480 | 0.4990 |
| Other bone disease and musculoskeletal deformities | 6 | 23.08% | 1.1756 | 0.4217 | 3.2771 | 0.7570 |
| Cardiac and circulatory congenital anomalies | <5 |  |  |  |  |  |
| Genitourinary congenital anomalies | <5 |  |  |  |  |  |
| Joint disorders and dislocations; trauma-related | 8 | 16.33% | 0.8507 | 0.3503 | 2.0661 | 0.7210 |
| Fracture of neck of femur (hip) | 97 | 18.13% | 0.6476 | 0.3889 | 1.0784 | 0.0950 |
| Spinal cord injury | <5 |  |  |  |  |  |
| Skull and face fractures | <5 |  |  |  |  |  |
| Fracture of upper limb | 38 | 14.79% | 0.6605 | 0.3718 | 1.1734 | 0.1570 |
| Fracture of lower limb | 41 | 12.39% | 0.5573 | 0.3169 | 0.9799 | 0.0420 |
| Other fractures | 25 | 10.82% | 0.4284 | 0.2306 | 0.7956 | 0.0070 |
| Sprains and strains | <5 |  |  |  |  |  |
| Intracranial injury | 33 | 32.35% | 1.6014 | 0.8613 | 2.9772 | 0.1370 |
| Crushing injury or internal injury | 14 | 25.93% | 1.3815 | 0.6422 | 2.9719 | 0.4080 |
| Open wounds of head; neck; and trunk | <5 |  |  |  |  |  |
| Open wounds of extremities | <5 |  |  |  |  |  |
| Complication of device; implant or graft | 49 | 23.11% | 1.0173 | 0.5814 | 1.7800 | 0.9520 |
| Complications of surgical procedures or medical care | 45 | 34.09% | 1.8838 | 1.0503 | 3.3788 | 0.0340 |
| Superficial injury; contusion | <5 |  |  |  |  |  |
| Burns | <5 |  |  |  |  |  |
| Poisoning by psychotropic agents | 12 | 35.29% | 2.4528 | 1.0540 | 5.7076 | 0.0370 |
| Poisoning by other medications and drugs | 6 | 20.00% | 0.9061 | 0.3303 | 2.4857 | 0.8480 |
| Poisoning by nonmedicinal substances | <5 |  |  |  |  |  |
| Other injuries and conditions due to external causes | <5 |  |  |  |  |  |
| Syncope | 7 | 18.42% | 0.6847 | 0.2666 | 1.7582 | 0.4310 |
| Fever of unknown origin | 11 | 22.45% | 1.0432 | 0.4610 | 2.3606 | 0.9190 |
| Lymphadenitis | <5 |  |  |  |  |  |
| Gangrene | 37 | 50.00% | 2.9329 | 1.5341 | 5.6069 | 0.0010 |
| Shock | <5 |  |  | | | |
| Nausea and vomiting | <5 |  |  |  |  |  |
| Abdominal pain | <5 |  |  |  |  |  |
| Allergic reactions | <5 |  |  |  |  |  |
| Rehabilitation care; fitting of prostheses; and adjustment of devices | <5 |  |  |  |  |  |
| Administrative/social admission | <5 |  |  |  |  |  |
| Medical examination/evaluation | <5 |  |  |  |  |  |
| Other aftercare | 52 | 20.39% | 0.8858 | 0.5106 | 1.5366 | 0.6660 |
| Other screening for suspected conditions (not mental disorders or infectious disease) | 9 | 9.09% | 0.3375 | 0.1478 | 0.7706 | 0.0100 |
| Residual codes; unclassified | 20 | 28.99% | 1.6509 | 0.8227 | 3.3128 | 0.1580 |
| Adjustment disorders | 9 | 31.03% | 1.9991 | 0.8010 | 4.9893 | 0.1380 |
| Anxiety disorders | 7 | 46.67% | 3.9049 | 1.2752 | 11.9577 | 0.0170 |
| Attention-deficit, conduct, and disruptive behavior disorders | 14 | 42.42% | 3.2028 | 1.3926 | 7.3658 | 0.0060 |
| Delirium, dementia, and amnestic and other cognitive disorders | 15 | 23.08% | 0.9565 | 0.4568 | 2.0028 | 0.9060 |
| Developmental disorders | <5 |  |  |  |  |  |
| Mood disorders | 64 | 27.59% | 1.7537 | 1.0166 | 3.0251 | 0.0430 |
| Personality disorders | 8 | 47.06% | 3.9740 | 1.3754 | 11.4822 | 0.0110 |
| Schizophrenia and other psychotic disorders | 46 | 18.93% | 1.0057 | 0.5723 | 1.7673 | 0.9840 |
| Alcohol-related disorders | 46 | 30.87% | 1.8072 | 1.0145 | 3.2191 | 0.0450 |
| Substance-related disorders | 16 | 29.63% | 1.7504 | 0.8265 | 3.7069 | 0.1440 |
| Suicide and intentional self-inflicted injury | 9 | 34.62% | 2.2774 | 0.8958 | 5.7897 | 0.0840 |
| Miscellaneous mental health disorders | <5 |  |  |  |  |  |

Notes: ED – emergency department. Min – Minimum (lower limit of 95% confidence interval). Max – Maximum (upper limit of 95% confidence interval). OR – odds ratio. Ref. – reference group.
